# A Review on Friction Stir Welding of Copper: Tool Geometry, Process Parameters, and Joint Properties

**DOI:** 10.3390/ma17215374

**Published:** 2024-11-03

**Authors:** Răducu Nicolae Bulacu, Matthieu Dhondt, Younes Demmouche, Claudiu Bădulescu, Eduard Laurențiu Nițu, Daniela Monica Iordache

**Affiliations:** 1 Faculty of Mechanics and Technology, National University of Science and Technology Politehnica Bucharest, 060042 Bucharest, Romania; raducu.bulacu@upit.ro (R.N.B.); eduard.l.nitu@upb.ro (E.L.N.); 2 Dupuy de Lôme Research Institute (IRDL)—UMR CNRS 6027, ENSTA Bretagne, F-29200 Brest, France; matthieu.dhondt@ensta-bretagne.fr (M.D.); younes.demmouche@ensta-bretagne.fr (Y.D.); claudiu.badulescu@ensta-bretagne.fr (C.B.)

**Keywords:** friction stir welding (FSW), copper, tool geometry, microstructure, mechanical properties

## Abstract

This paper comprehensively reviews friction stir welding (FSW) as applied to copper and its alloys. FSW is a solid-state joining process that offers significant advantages over traditional fusion welding methods, particularly for materials like copper that are difficult to weld conventionally due to their high thermal conductivity and oxidation issues. Over time, the FSW process has been developed for different industries. Copper structures joined through FSW are utilized for nuclear waste storage, electrical connectors, chemical and petrochemical storage, refrigeration systems, heat exchangers, and the aerospace industry. This covers recent advancements in FSW technology, the geometry of the tools used, the process parameters, and the microstructural characteristics and mechanical properties of the joints. It examines the shapes, sizes, and materials of the tools used for welding copper and its alloys, along with process parameters such as rotational speed and traverse speed, and their influence on the quality of the joints. Additionally, the paper presents syntheses of previously published results, highlighting the values of parameters that indicate the quality of the welds, including grain size, microhardness, mechanical strength, and elongation. The challenges and potential solutions in applying FSW to copper are also discussed, providing a starting point for future research and industrial applications.

## 1. Introduction

Copper and its alloys are widely used in industry as they have advantageous properties such as high thermal and electrical conductivity, good mechanical and corrosion resistance, good ductility, etc. [1,2]. Due to its high thermal conductivity and high oxidation rate, copper is difficult to weld by conventional welding processes [3]; over the last 15 years, a great deal of research has been carried out on joining copper and its alloys by friction stir welding (FSW).

**Figure 1:**
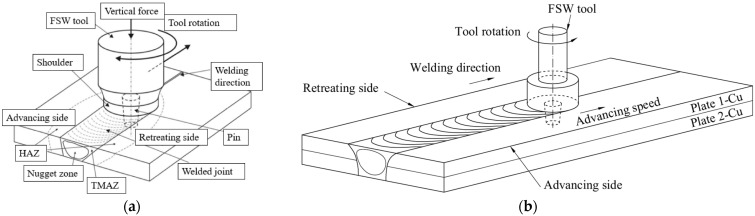
Schematic of the FSW process: (**a**) butt joint [12]; (**b**) lap joint.

Friction stir welding is a solid-state welding process developed and patented by W.M. Thomas et al. at The Welding Institute (TWI, UK) in 1991 [4]. According to the literature [3,5,6,7,8,9,10,11], FSW has the following advantages: FSW-welded structures have reduced distortion and shrinkage; FSW joints have superior mechanical properties and a fine microstructure; no filler material is needed; there is no need for gasses or shielding materials; the process has low energy consumption; there is no molten material, so no spatter or other gasses are formed; there is no loss of alloying elements; no UV radiation; and no post-weld mechanical processing required. There are several FSW process schemes, which differ in the positioning of the plates, the input parameters used, the technological role of the parts in the ex-welding, etc. The most widely used FSW process schemes are shown in Figure 1. Figure 1a represents a butt positioning of the plates, where the joint is realized between the common side surfaces, and Figure 1b shows the stacked positioning of the plates, where the joint is realized between the lower surface of the upper plate and the upper surface of the lower plate.

FSW is a solid-state joining method in which a special rotating tool, consisting of a shoulder and a pin, moves along the contact surfaces of plates rigidly fixed to a backing plate. The shoulder presses against the upper surfaces of the plates and the pin mixes the volume of material. As the tool moves along the welding direction, the material of the plates undergoes severe plastic deformation and is transported from the front of the tool to the back.

**Figure 2:**
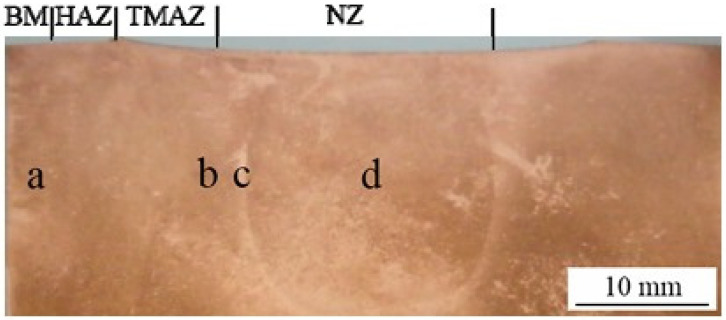
Microstructural regions of the FSW process for pure Cu 1/2H [14]. (a) base metal (BM); (b) heat affected zone (HAZ); (c) thermo-mechanically-affected zone (TMAZ); (d) nugget zone (NZ).

The friction between the shoulder of the tool and the top surface of the plates, respectively, and the friction between the tool pin and the splice materials produce heat and severe plastic deformation leading to the material being brought into a visco-plastic state and the weld bead forming. As can be seen in Figure 2, the weld bead produced by FSW is divided into four distinct zones: the nugget zone (NZ), the thermo-mechanically affected zone (TMAZ), the heat-affected zone (HAZ), and the base material (BM), the unaffected zone [13].

Over the years, the FSW process has been developed according to several principles and efficiency strategies for different fields of use. In terms of the exploitation of FSW-joined copper structures, these are found in various fields: tanks and canisters for nuclear waste [13,15,16,17], electrical connectors [18,19,20], chemical and petrochemical fuel tanks [17,21,22], heat exchangers [18], the aerospace industry [21,23,24], and refrigeration installations [19,25,26].

This paper analyzes the results obtained by the friction stir welding of copper and its alloys in terms of microstructure, mechanical properties, and the temperatures obtained, as published in the literature. The shapes, sizes, and materials of the tools used for welding copper and its alloys, as well as the technological parameters (rotational speed and traverse speed) and their influence on the quality of the weld, were analyzed. Also presented are summaries based on the results published so far on the values of the parameters denoting the quality of the joints obtained (grain size, microhardness, mechanical strength, elongation).

To fully understand the effects of the main process parameters, such as rotational speed, traverse speed, penetration force, as well as the shape, dimensions, and tilt angle of the tool on the microstructure and mechanical properties of the welded joints, it is necessary to analyze the joints obtained under different conditions. The most important factors that appear during the welding process are the heat generated by the friction between the FSW tool and the weld plates and the plastic deformation of the weld material.

This paper gives an overview of the current state of research in the field of FSW of copper and its alloys, focusing on the positive and negative aspects of this process. A synthesis of information from previous studies is presented, which is particularly useful to researchers in the field, as it provides recommendations for the improvement of technological parameters and tool geometry in order to obtain defect-free joints with superior mechanical properties suitable for various industrial applications. The syntheses presented and the conclusions drawn can form the basis for future fundamental and applied research in an efficient and well-documented manner.

## 2. Tool Geometry

The tool used in the FSW process realizes rotational and traverse motion and heats the materials to the optimum temperature, plastically deforms the base materials, and mixes them to produce a high-quality bead. The geometry and dimensions of the FSW tool have an important impact on the quality of the resulting joints. The tool must have a specific geometry to provide an even temperature distribution and proper material flow. The correct choice of tool geometry can lead to flawless packings with superior mechanical properties and a uniform microstructure.

The construction of the tool comprises a shoulder, which retains the mixed material and provides thermal input by friction with the upper surfaces of the mixing plates, and a pin which penetrates the plates and generates heat by local plastic deformation and mixes the material. The shoulder can have various shapes such as: flat [6,8,14,15,21,27,28,29,30,31,32,33,34,35,36,37,38,39,40,41,42,43,44,45,46,47,48], concave [1,13,16,29,49,50,51,52,53,54], spiral [29,55].

When welding pure copper, the flat shoulder, concave shoulder, and spiral shoulder have different behaviors in terms of heat generation and material mixing. The flat shoulder is simple, easy to achieve, but can generate more heat in a certain area, which can lead to overheating of the material and a higher risk of defect formation. In contrast, the concave shoulder leads to a more even heat distribution and has frequently been used when welding 3 mm thick plates. Thus, when welding 3 mm thick Cu plates at low rotational speeds and traverse speeds of the order of 50 mm/min–100 mm/min, higher quality welds with improved mechanical properties and finer microstructure are obtained [49,50,51,56]. The spiral shoulder enables more precise temperature control during welding. By distributing the heat evenly, this shape helps maintain a constant temperature in the weld zone, preventing overheating and degradation of the microstructure of the material [51]. However, when using technological parameters that lead to excessive heating (800 °C at low rotational and traverse speeds), the inferior mechanical properties of the base material are obtained [55].

**Figure 3:**
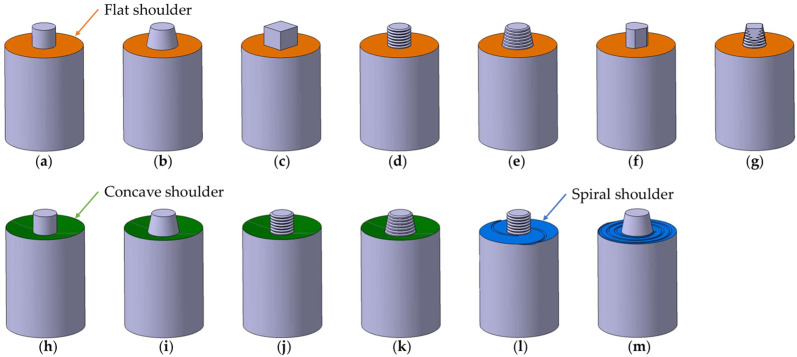
FSW tool shapes used for copper butt joints: (**a**) flat shoulder (orange) and cylindrical pin; (**b**) flat shoulder and conical pin; (**c**) flat shoulder and square pin; (**d**) flat shoulder and cylindrical threaded pin; (**e**) flat shoulder and conical threaded pin; (**f**) flat shoulder and cylinder with three-plane pin; (**g**) flat shoulder and threaded cylindrical pin with four planes; (**h**) concave (green) shoulder and cylindrical pin; (**i**) concave shoulder and conical pin; (**j**) concave shoulder and threaded cylindrical pin; (**k**) concave shoulder and conical threaded pin; (**l**) spiral shoulder (blue) and threaded cylindrical pin; (**m**) spiral shoulder and conical pin.

In the literature, the pin is found in different forms, the main ones being: a cylindrical pin [1,7,13,30,34,35,38,39,40,44,45,46,47,57,58,59], a cylindrical threaded pin [3,6,14,15,16,21,29,31,32,36,37,38,41,42,48,50,51,52,53], a conical pin [8,27,28,43,55,56,60,61,62,63,64], a conical threaded pin [28,33,49,54,65], and a square pin [32,34]. Figure 3 represents the tool shapes of FSW (shoulder and pin) tools used for copper butt joints based on the literature [1,3,6,7,8,13,14,15,16,21,27,28,29,30,31,32,33,34,35,36,37,38,39,40,41,42,43,44,45,49,50,51,52,53,54,55,56,57,64,65,66,67].

The cylindrical pin is commonly used because it facilitates uniform heat distribution, has a simple shape, and is easy to obtain, but it can lead to voids and pores when joining thick sheets due to low material flow. The threaded cylindrical pin reduces the risk of voids and pores, providing a higher quality weld, but can generate additional heat, requiring careful control of welding parameters to prevent the overheating of the material. The conical pin facilitates penetration and is often used for thicker materials or copper alloys. In the case of threaded conical pins, the threading improves material mixing around the pin. The square shape of the pin helps to create shear forces, which improves material mixing and reduces the risk of flaw formation but can lead to faster pin wear due to large contact surfaces and high forces.

The flat shoulder of the tool is generally between Ø12 mm and Ø24 mm in diameter, the associated pin being cylindrical (Ø3–Ø8 mm), with a cylindrical thread (M3–M8), and a conical or square cross-section depending on the thickness and type of material used. The concave shoulder has diameters ranging from Ø12 mm to Ø20 mm and is generally conical, threaded conical, or threaded cylindrical pin.

**Figure 4:**
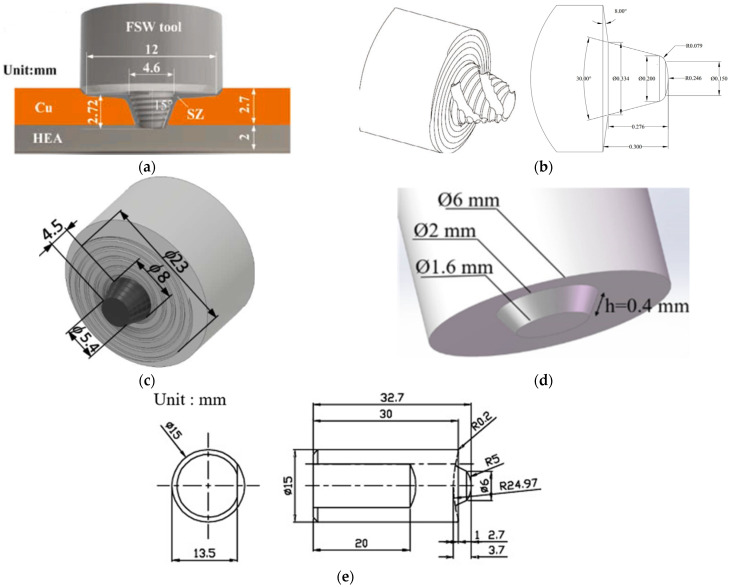
Schematic representations of FSW tools: (**a**) threaded conical pin [68]; (**b**) spiral conical pin featuring helical grooves [69]; (**c**) threaded conical pin [55]; (**d**) conical pin [70]; and (**e**) conical pin [71].

Figure 4 shows graphical representations of the FSW tools used for joining copper or copper alloys with different plate thicknesses. Specifically, Figure 4a shows a tool with a flat shoulder and threaded conical pin used for lap welding between Cu T3 and a multicomponent HEA alloy. Figure 4b illustrates a tool with a spiral shoulder and a spiral conical pin featuring helical grooves, which join the copper rotor’s end rings. Figure 4c represents a tool with a flat shoulder and a threaded conical pin used for butt-joining Cu-ETP R220 plates with a thickness of 5 mm.

Figure 4d is a tool with a flat shoulder and conical pin used for joining ultra-thin sheets of Cu T2 and H62 brass with a thickness of 0.6 mm. Figure 4e represents a tool with a concave shoulder and conical pin used for welding Beryllium-Copper alloy C17200 with a plate thickness of 3 mm.

Xue et al. [15] used a Ø20 mm flat shoulder tool with a 4.7 mm long M6 threaded cylindrical pin and obtained defect-free joints when welding two pure copper plates butt at low heat input, i.e., for rotational speeds of 400–800 rpm and traverse speed of 50–200 mm/min. Using the same tool geometry, Xie et al. [31] achieved good quality joints with reduced heat input.

Wang et al. [67] used a tool with a concave shoulder and threaded conical pin when welding a harder copper alloy where he obtained defect-free joints and sufficient material flow even though the heat input was not the greatest.

Khodaverdizadeh et al. [32] compared the results obtained when using a square pin and a threaded cylindrical pin, the other tool characteristics being identical, in which it was found that using the square pin resulted in much higher quality and stronger structures compared to the threaded cylindrical pin. Also, Galvão et al. [29] affirms that for tools with a flat shoulder, the FSW tool’s torque is much lower than that of a concave or spiral shoulder, but provides much poorer material flow, resulting in flawed structures, whereas the spiral shoulder provides adequate material flow and higher grain refinement. The spiral-shouldered tool generated the most heat and favored optimal material flow, resulting in higher quality joints.

The most frequently used FSW tooling materials for butt welding of copper structures are high-speed steels H13 [3,30,34,35,36,37,39,43,53]. Tungsten carbide tools were used for the thicker plate WC [33], and for high-hardness copper alloys, alloys based on Ni [49], Inconel 718 [65], TiC-based cermet [67], X210Cr12 [64], X32CrMo3 3 [42], X32CrMoV12-28 [6], and SKD61 [72].

H13 high-speed steels are low-cost and have good hardness but wear quickly when welding copper due to the high thermal conductivity of copper. Metallic carbides have good wear resistance and thermal stability and can be used at high temperatures without degradation, but are brittle and expensive. Nickel-based alloys have good thermal stability, oxidation resistance, and maintain their mechanical properties at high temperatures, but are expensive and difficult to machine. Composite materials have very good hardness and wear resistance and superior mechanical and thermal stability, but are complex and costly to manufacture.

## 3. Process Parameters of FSW

The most important parameters of the FSW are the rotational speed of the tool, which can be clockwise or counterclockwise, and the traverse speed, which can be either the tool or workpiece traverse speed. The quality of the joints is influenced by the combination of rotational speed and traverse speed. The best combination should ensure good mixing of the material, prevention of defects, and superior mechanical properties. According to the literature, the range of tool speed values varies from 200 to 2050 rpm and the traverse speed is in the range of 16–900 mm/min, which denotes a fairly high flexibility in adapting the FSW process to different copper alloys and operating conditions.

The most used values of the technological parameters of the FSW process are a rotational speed of 600 rpm [3,7,15,21,28,31,32,34,36,38,39,46,49,50,51,67] and a traverse speed of 50 mm/min [3,7,15,31,35,39,49,51,52,67]. The tilt angle of the tool has generally been in the range of 1–3°, but most investigations have been carried out without tilting the tool [3,6,8,19,28,30,31,32,35,36,39,40,42,43,47,48,49,50,51,53,55,57,58,63,65,66,73]. The most used tool angle was 2.5° [17,23,34,38,41,44,52,54,72].

Sun et al. [7] studied the effects of rotational speed on the microstructure and mechanical properties of Cu T2 joints by keeping the traverse speed constant at 50 mm/min and varying the rotational speed for each experiment. They obtained the best results when using a rotational speed of 1000 rpm. At this rotational speed, a fine microstructure without defects and with superior mechanical properties was obtained. The same strategy has been addressed by Liu et al. [50], which for a traverse speed of 100 mm/min identified a rotational speed of 400 rpm as the best option for pure copper 1/2H joining. At rotational speeds above 600 rpm, larger grain sizes resulted in the bead zone due to the temperature rise and plastic deformation, while the grain size in the HAZ is not influenced by the increase in rotational speed.

The comparison of the parameters was also approached by keeping the rotational speed constant, where Shen et al. [51] found that most defects occurred at higher traverse speeds. It was observed that defects occurred at high traverse speeds (above 200 mm/min) and that the weld bead size decreased with increasing traverse speed. It has been established that the best traverse speed for FSW of pure copper in combination with a rotational speed of 600 rpm is about 50–100 mm/min, which achieves superior mechanical properties and a fine microstructure. Also, Barenji [34] achieves better properties than the base material by using a rotational speed of 600 rpm, and in cases where this is higher, the quality of the joint properties decreases significantly. The defects occurred at high traverse speeds and low rotational speeds. The tool used had a square pin and the heat input was empirically calculated as a function of the welding parameters, with the result that high rotational speeds and low traverse speeds increase the heat input.

The tool tilt angle aids in controlling material flow and generating frictional heat, facilitating material mixing and improving weld quality. Consequently, it helps reduce defects by ensuring a more uniform heat distribution in the welded area. However, the tilt angle requires precise tool control to prevent deviations and may contribute to uneven tool wear.

A tool tilt angle of 2.5° was used by Xue et al. [15] to weld 1/2H pure copper with a thickness of 5 mm, employing a flat-shouldered tool with a threaded cylindrical pin. They measured mechanical properties very close to those of the base material. The tool tilt contributed to material homogenization in the nugget zone (NZ), preventing void formation and enhancing the overall hardness of the welded area. The tool inclination facilitated the movement of the material from the advancing side to the retreating side, preventing the formation of voids or insufficiently mixed regions. Using similar parameters and the same tool design, Xie et al. [31] achieved mechanical properties comparable to those of the base material without tilting the tool. Defect-free joints were obtained, although the hardness achieved with similar welding parameters was lower.

A tilt angle of 2.5° used with different tool configurations shows an important influence on material flow and temperature distribution. For instance, the use of a concave shoulder Ø14 mm with a cylindrical threaded pin Ø4 mm at a tilt angle of 2.5° with rapid cooling significantly improved joint hardness and mechanical properties, particularly when welded at 400 rpm and 50 mm/min. The rapid cooling technique in conjunction with the tilt angle helped manage the excessive heat that could otherwise degrade joint quality [52].

The tilt angle can help in tailoring the FSW process to different material thicknesses and properties. For example, with a flat shoulder Ø20 mm and square pin 4.3 × 4.3 mm, a tilt angle of 2.5° at rotational speeds between 600 and 1200 rpm, and traverse speeds from 25 to 100 mm/min, resulted in different temperature profiles. This shows that the tilt angle, combined with specific rotational and traverse speeds, can be optimized to enhance joint quality [34].

A summary of published results on the FSW of copper is shown in Table 1, while results on the FSW of copper alloys are presented in Table 2. The two tables offer detailed information on FSW tooling, including the geometrical shape and material, as well as the process parameters used in the butt joining of plates. Each table focuses on different material categories and is organized by increasing plate thickness. In these tables, Vr represents the rotational speed, Va indicates the traverse speed, and Fa denotes the penetration force.

The table presents variations in tooling, including shoulder type, pin geometry, materials used, and key process parameters like rotational speed, traverse speed, and applied force. These factors influence heat generation, material flow, and grain structure, directly impacting the mechanical properties and quality of the welded joint.

Table 2 focuses on copper alloys, which require different approaches than pure copper. Each entry provides details about the friction stir welding (FSW) tooling, including shoulder type, pin design, and the materials used for the tools, as well as specific process parameters tailored to the properties of copper alloys. Copper alloys, which may include elements like zinc, chromium, and zirconium, behave differently during the FSW process, often resulting in distinct microstructural and mechanical changes compared to pure copper. For example, the presence of these alloying elements can lead to grain refinement and increased hardness in the nugget zone (NZ). Additionally, adjusting rotational and traverse speeds can enhance control over these outcomes.

The following conclusions can be deduced from the summary presented in Table 1:

- Tools with a flat or concave shoulder and cylindrical or threaded cylindrical pin can be used successfully when welding Cu plates by generating the heat required for joining;
- Regardless of the shape and size of the tool and the values of the technological parameters in the heat-affected zone (HAZ), a microstructure with coarse grains and the poorest mechanical properties are obtained due to incomplete recrystallization;
- The nugget zone (NZ) is usually finer-grained than the heat-affected zone (HAZ) due to complete recrystallization, which leads to higher hardness and strength in the welded zone than in the HAZ;
- A temperature of 550 °C during friction stir welding (FSW) for pure copper is considered ideal for preventing defects and achieving good mechanical properties;
- The material thickness of the joined plates influences the choice of optimal process parameters.

Lower rotational speeds (400–600 rpm) combined with a medium traverse speed (50–100 mm/min) have been used for welding copper plates thicker than 3 mm; good results have been obtained when welding 3 mm thick plates with a rotational speed of 900 rpm and a traverse speed of 40 mm/min; for smaller thicknesses, higher traverse speeds are used to avoid the overheating of the material.

The following conclusions can be drawn from the summary presented in Table 2:

- Zinc content influences grain size in the joint area and joint hardness; a higher zinc content leads to smaller grains and increased hardness;
- The Cu-Cr-Zr alloy joints have improved microstructure and mechanical properties by using post-welding treatments such as rapid water cooling;
- For thin materials, higher rotational speeds (700–1000 rpm) and high traverse speeds are often more effective in ensuring homogeneous mixing and optimal temperature; for thicker materials, rotational speeds and traverse speeds should be reduced to allow optimal heating and prevent material damage;
- Defects such as porosities, cracks, and tunnels occurred when process parameters were incorrectly chosen.

The butt joining of copper and its alloys by the FSW process has become a topic of interest due to properties such as corrosion resistance, good thermal conductivity, and electrical conductivity.

In the FSW process, process parameters (tool rotation speed, traverse speed) control the amount of heat generated by friction and plastic deformation, which leads to a rise in temperature during the process. The temperature during friction stir welding influences the microstructure and mechanical properties of the weld as a consequence of the chosen process parameters.

Experimental research has focused on optimizing the process parameters to obtain microstructures leading to joints with better mechanical properties than those of the base material, free from defects, using both traditional and hybrid FSW processes.

## 4. Process Temperature

The temperature in the FSW process is an important factor that can be optimized depending on tool geometry and process parameters [1,8,16,27,32,34,40,55,74]. Accurate temperature control leads to a uniform microstructure, defect prevention, and the superior mechanical properties of the welded joints. Research has been carried out to identify the optimum temperature range to obtain a joint without defects, such as cracks, pores, or voids, and with good mechanical properties. These studies aimed to establish the temperature at which the base material effectively bonds to form a homogeneous microstructure, the synthesis of which is shown in Table 3.

Hwang et al. [1] concluded that the optimum temperature for welding pure copper C11000 is in the range 460–530 °C, with the temperature on the advancing side being slightly higher than on the retreating side. Mironov et al. [19] studied the microstructure evolution of pure 1/2H copper during the FSW process. They measured temperatures ranging from 170 to 700 °C where they found that, for temperatures lower than 0.5 of the melting temperature (0.5 × Tm), the bead exhibited mostly continuously recrystallized granular structures, and for temperatures higher than 0.5 of the melting temperature, the granular structures were discontinuously recrystallized.

Khodaverdizadeh et al. [32] investigated the influence of the FSW tool pin shape on the microstructure and mechanical properties of pure copper joints produced by FSW. They found that the temperatures recorded during welding with a square pin were higher compared to those obtained using a threaded cylindrical pin. Barenji [34] measured maximum temperature values ranging from 380 to 492 °C for which he obtained defect-free joints, even at a lower heat input for pure copper with a thickness of 5 mm. Gheisari et al. [75] analyzed defect formation in FSW welded copper structures and measured a temperature of about 500 °C at a distance of 6 mm from the bond line of the plates.

Mironov et al. [40] concluded that, in the case of Cu-30Zn alloys, the joints are not qualitative when the temperature is below 0.46 of the melting temperature of the material, but for temperatures of 0.6–0.7 of the melting temperature, the welded structures improve considerably in quality, and at 0.8 of the melting temperature, the joints show voids at the base of the bead.

Jha et al. [8] measured the temperature during the FSW process at 2 mm, respectively, 12 mm from the shoulder of the tool on both sides of the plates (AS and RS) as about 800 °C, which is about 0.8 of the melting temperature, much too high for the CuCrZr alloy.

**Figure 5:**
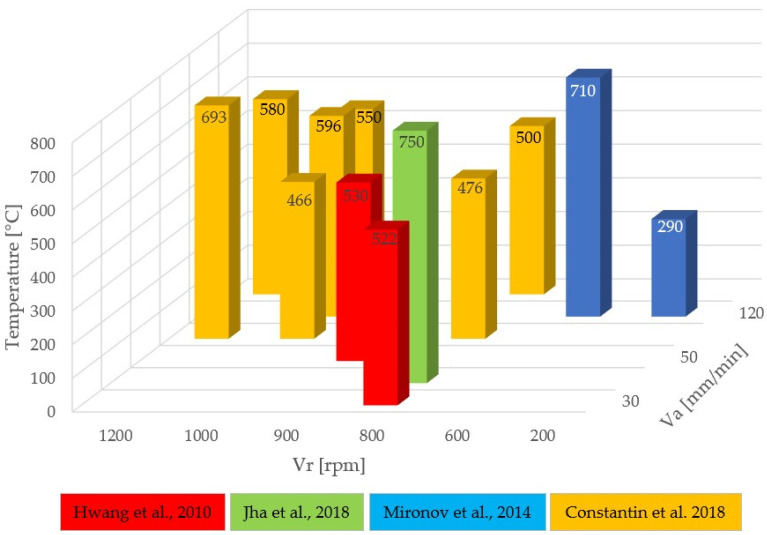
Influence of welding parameters on temperature distribution: red [1]; green [8]; blue [40]; yellow [63].

Based on analysis of the previously presented papers, it is clear that process temperatures vary significantly depending on both the rotation speed and the advance speed. For instance, in the friction stir welding of approximately 3 mm plates, the relationship between temperature and welding parameters is illustrated in Figure 5.

The temperatures obtained for defect-free joining of pure copper are lower than those for copper alloy joints. At a rotational speed of 800 rpm, temperatures between 476 °C and 522 °C are achieved for pure copper, depending on the feed rate. For pure copper, a temperature range of 460–550 °C has proven ideal for producing defect-free FSW joints with adequate hardness and mechanical strength. Values below 460 °C do not provide sufficient plasticization, while values above 550 °C may lead to coarse-grained structures, negatively affecting joint hardness. In the case of welding a Cu-30%Zn alloy, at a rotational speed of 600 rpm, a temperature of 710 °C resulted in discontinuous recrystallization with a coarse grain structure, leading to low hardness. At lower rotational speeds (200 rpm), the temperatures were insufficient, causing defects due to incomplete heating. For alloy joining at a temperature of 750 °C, achieved with a rotational speed of 800 rpm and a feed rate of 40 mm/min, defect-free joints were obtained.

Constantin et al. [63] examined the temperature during the FSW process of pure copper, focusing on its close relationship with process parameters and the formation of defects. Table 4 presents the average values of temperatures measured near the FSW tool for 3 mm thick Cu-DHP, along with the welding process parameters (feed rate and rotational speed) to evaluate the visual morphological aspect of the welded surfaces. Temperatures during the welding process ranged from 466 °C to 693 °C, representing 44% to 64% of copper’s melting temperature. A channel defect appears at the beginning of the joint in all cases, caused by insufficient material plasticization due to low temperatures or improper welding equipment settings. As temperature stabilizes, the visual quality of welded surfaces improves significantly. Research findings suggest that to prevent defects during the friction stir welding of pure copper plates, the welding temperature should be maintained between 550 °C and 650 °C [63]. In this range, the material is plasticized adequately to ensure effective mixing, preventing significant channel and burr-type defects. When temperatures exceed 650 °C, burr formation occurs due to excessive plasticization.

Mironov et al. [16] produced welded joints on 1/2H pure Cu plates with a thickness of 4 mm, maintaining a constant traverse speed of 2 mm/s (120 mm/min) for all cases. They achieved temperatures below 0.5 of copper’s melting temperature when the rotational speed ranged from 200 to 500 rpm, where continuous recrystallization was predominantly observed, resulting in a fine-grained microstructure (1–2 μm) in the nugget zone (NZ). For rotational speeds of 600 to 1000 rpm, the temperature exceeded 0.5 of the melting temperature, and discontinuous recrystallization became predominant, leading to a coarse grain structure (10–30 μm) and material softening.

Wang et al. [49] studied strategies for reducing the welding temperature below the aging temperature of a Cu-Cr-Zr alloy with a thickness of 7 mm. The peak temperatures achieved were 830 °C, 711 °C, and 481 °C, respectively. In the first two cases, the temperatures were very high, resulting in a much coarser grain structure compared to the case with the lower temperature. Regarding the microhardness distribution, in the first two cases the microhardness values were below those of the aged Cu-Cr-Zr alloy, while in the third case, the microhardness values exceeded the aging threshold of the material.

In light of the studies carried out, the following conclusions can be drawn regarding the temperature in the friction stir welding (FSW) process for copper and its alloys:

- The temperature in the FSW process influences the recrystallization mechanisms by determining the grain size, grain size distribution in the grain-binding zone, and the homogeneity of the microstructure;
- Higher than optimal temperatures lead to grain growth and decreased mechanical properties;
- For pure copper, the optimum temperature varies between 380 and 600 °C, with most studies recommending around 540–550 °C, i.e., 0.5 × Tm (copper melting temperature), as the optimum welding temperature;
- Copper alloys have a higher maximum temperature during the FSW process (0.8 × Tm) than pure copper.

## 5. Microstructure

Most of the studies on the microstructure of the joint have been oriented towards the analysis of the microstructure’s evolution in different zones and the analysis of the recrystallization mechanisms as a function of process parameters. Analysis of microstructure in the heat-affected zones and the central zone of the joint includes the study of grain size, the existence of defects, and microstructural texture to understand how the FSW process affects the microstructure of the base material.

In a series of research studies [7,13,14,21,32,34,38,41,45,50,51,64,65,66] it was established that during the FSW process, the microstructure of the base material changes substantially and leads to the emergence of the weld core zone (NZ), the thermally affected zone (HAZ), and the thermo-mechanically affected zone (TMAZ).

Liu et al. [50] investigated the effect of tool rotational speed on the microstructure and mechanical properties of pure copper 1/2H FSW joints, keeping the traverse speed unchanged. They concluded that by increasing the tool rotation speed, the grain size in the NZ and TMAZ increased and the boundary between these zones was blurred. As for the HAZ, the rotational speed had almost no effect on grain size.

Shen et al. [51] investigated the effect of traverse speed on the microstructure and mechanical properties of FSW joints of pure copper 1/2H. Their results show that with increasing traverse speed, the grain size in the NZ decreased, while the TMAZ became much smaller and harder to identify, and the HAZ showed very small changes in grain size.

**Figure 6:**
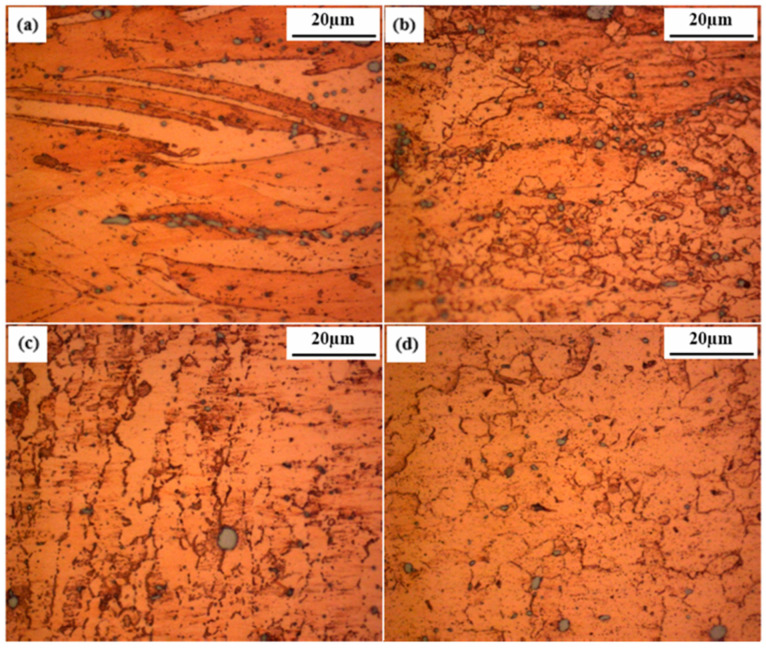
Microstructure of CuCrZr alloy joints: (**a**) base material; (**b**) heat-affected zone; (**c**) thermo-mechanically affected zone; (**d**) nugget zone [14].

Figure 6 shows that the microstructure of the base material is coarse, while the HAZ exhibits a granular structure similar to that of the base material. The TMAZ is a highly deformed zone, with material flow directed toward the NZ, which is much finer than the other zones due to the high temperatures generated by friction and the plastic deformation of the copper alloy.

### 5.1. Nugget Zone

During the FSW process, a fine-grained microstructure is found in the NZ due to recrystallization as a result of plastic deformation and frictional heat. Some researchers have obtained onion ring microstructures in the nugget zone [8,13,15,16,31,43,45,50,54,56].

Barenji [34] studied the influence of heat input on the microstructure evolution and mechanical properties of copper joints made by FSW. He obtained joints with fine and equiaxed grains. The grain size increased with decreasing traverse speeds at a constant rotational speed and with increasing rotational speeds at a constant traverse speed. Thus, the author demonstrated that the weld core (NZ) grain size is mainly affected by the strain rate and maximum temperature during the FSW process. Table 5 shows the NZ grain sizes as a function of the nature and thickness of the material, the tool geometry, and the welding parameters used for different types of copper alloys. In general, for thin materials (1–2 mm), the grain size tends to be smaller because heat transfer is more efficient, and the material is easier to deform plastically. In materials with thicknesses of 2.5–3 mm the hole size shows a larger variation, being strongly influenced by process parameters. In the case of thicker plates (4–7 mm) the grain size tends to be larger, but can be controlled by the appropriate choice of pin shape and process parameters. Alloys with a high zinc content tend to have smaller grains compared to pure copper, due to the refining effect induced by zinc.

Yaghoubi et Shirazi [21] analyzed the initiation zones, stable growth zones, and unstable crack growth zones for the NZ by scanning electron microscopy (SEM) method under the conditions of a rotational speed of 600 rpm and an advancement speed of 40 mm/min. They observed that several cracks occur during crack initiation that grow to fatigue failure due to the presence of fatigue lines and striations, but pits also occur involving ductile cracking of the material that may be produced unstably or abruptly, in which case the fracture is not brittle. Using transmission electron microscopy (TEM), Heidarzadeh et al. [30] analyzed the NZs of joints between pure copper and brass. They observed that brass exhibits much larger dislocations than pure copper, copper exhibits cell-like dislocations, and in the case of brass the dislocations are entangled structures. We can say that the recovery of dislocations occurs in copper due to its higher energy, which in the case of brass inhibits the transverse sliding of dislocations.

### 5.2. Thermo-Mechanically Affected Zone

The TMAZ is the zone in which the material has suffered significant plastic deformation and has been exposed to heat, but not enough to produce complete recrystallization. In general, the grains in this zone are elongated and deformed, larger in size than those in the nugget zone.

Barenji [34] affirms that the TMAZ exhibits elongated grains that are larger than those of the NZ, but smaller than those of the base material. By increasing the rotational speed, the TMAZ exhibits an equiaxial recrystallized grain structure due to the higher percentage of heat during FSW, which is similar to the grain structure of the NZ, which makes the boundary between the two zones blurred. Lai et al. [14] find that in the TMAZ there are high heating temperatures due to friction and plastic deformation, resulting in a strongly deformed structure with slip grains along the material flow direction. When welding pure copper plates, the TMAZ exhibits higher hardness and mechanical strength due to microstructure refinement and the formation of fine elongated grains, but compared to the NZ, the hardness and mechanical strength are lower [1,18].

### 5.3. Heat-Affected Zone

The HAZ is the zone that is affected by heat but does not undergo any plastic deformation. The material in this zone did not undergo pin or tool-shoulder friction, but was thermally influenced by the heat generated by the FSW process, leading to annealing and microstructural changes. Most studies to date have concluded that the microstructure of the HAZ exhibits large grain sizes, sometimes larger than those of the base metal. As far as the parameters of the welding regime are concerned, they do not influence the grain size in this zone much [34,50,51].

## 6. Mechanical Properties

Studies on the mechanical properties of FSW joints have focused on the evaluation of the tensile strength (UTS) and the elongation of the welded joints, comparative evaluation of the mechanical properties of the joints with those of the base material (the ratio representing the efficiency of the joint), analysis of the hardness distribution in different zones, the behavior of welded joints under cyclic stress, and analyzing the joints’ fatigue resistance.

The tensile strength and microhardness of the weld bead indicate the joint quality of FSW structures. The mechanical properties depend on the microstructure of the FSW seam. The examination of the microhardness distribution and the mechanical strength of the joints leads to the validation of the process parameters and, at the same time, to the validation of the adopted strategy. Table 6 summarizes the mechanical properties of Cu joints produced by the FSW process, obtained by different researchers, namely: ultimate tensile strength (UTS), yield strength (YS), microhardness, and elongation.

In addition, we calculated the link efficiency, E, with relationship (1):

(1) $$E=\frac{{UTS}_{FSW}}{{UTS}_{BM}}\cdot 100[\%]$$

where UTS_FSW_ is the ultimate tensile strength value of the specimen extracted from the FSW joint and UTS_BM_ is the ultimate tensile strength value of the base material.

### 6.1. Mechanical Strength

Researchers have focused on identifying the process parameters that lead to a mechanical strength of the material that is greater than or equal to that of the base material.

In the case of the welding of 7 mm thick Cu-Cr-Zr copper alloy plates treated in solid solution at 980 °C for 1.5 h, then rapidly cooled in air or water, Wang et al. [49] observed that the mechanical strength of the joints obtained for the supersaturated plates closely approximated those of the base material for rotation speeds of 30–600 rpm and traverse speeds of 50–100 mm/min. They also made plate joints for which, after solid solution treatment, the plates were aged at 480 °C for 2 h, resulting in mechanical properties far superior to the previous ones; for these, water cooling resulted in specimen breakage in the zone of the base material, while for air cooling the breakage was in the NZ. For the same Cu-Cr-Zr copper alloy, but with a thickness of 2.5 mm, Wang et al. [67] welded aged hot-rolled material plates using FSW, and they kept the traverse speed constant at 50 mm/min and varied the rotational speed between 400 and 600 rpm. They obtained a mechanical strength close to that of the base material by using a rotational speed of 400 rpm, and specimen fracture was in the zone of the base material, while for the other joints, the fracture was in the NZ.

For 5 mm thick pure copper, Khodaverdizadeh et al. [32] performed experiments using the same parameters for two tool configurations, one with a square pin and one with a threaded cylindrical pin. Conducting an analysis on the NZ, the actual joint produced with the square pin shows a higher strength than that obtained by using the threaded cylindrical pin, which is due to the much better material mixing achieved using the square pin. Regarding the welding of 2 mm thick pure Cu and Cu-30Zn plates, Wang et al. [43] made joints using the same tool and rotational speed for both materials, and the traverse speed was 100 mm/min for pure Cu and 200 mm/min for Cu-30Zn. For both materials, they obtained joints with strengths very close to those of the base materials, and the breakage of the specimens was realized in the zone of the base material in both cases, thus observing that by adding zinc to the copper alloy, for the same rotational speed, the traverse speed must be increased to obtain high-quality joints.

### 6.2. Microhardness

The studies consisted of assessing the hardness distribution in different joint zones, such as the heat-affected zone (HAZ), the thermo-mechanically affected zone (TMAZ), and the nugget zone (NZ). The aim was to understand how process parameters and microstructural variations influence the hardness in these zones and how this correlated with other mechanical properties.

**Figure 7:**
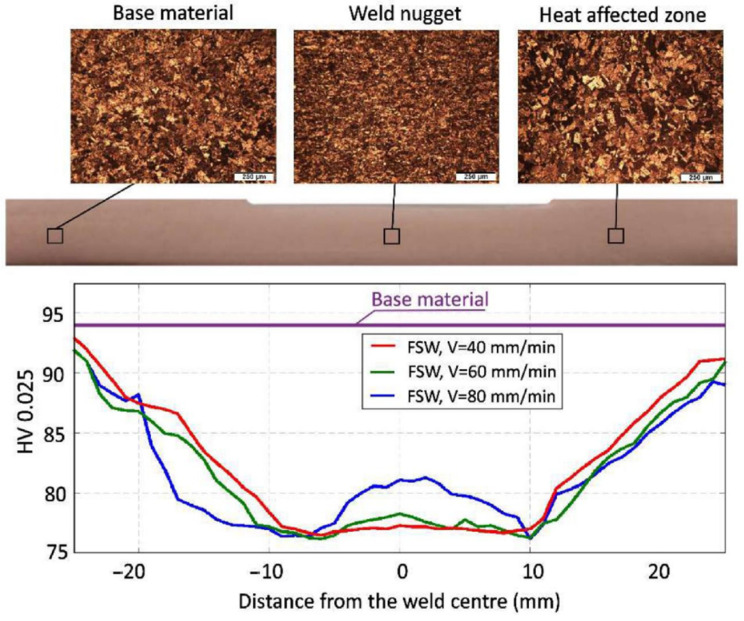
Microstructure and microhardness of Cu-ETP R220 [55].

In general, the microhardness profiles have a characteristic “W” shape, typical for FSW joints, a shape that indicates clear differences in hardness across the various zones of the welded joint: NZ, TMAZ, and HAZ, see Figure 7 [55]. Regardless of the traverse speed, the lowest microhardness was obtained in the heat-affected zone (HAZ), and higher traverse speeds lead to greater variations in microhardness due to lower heat input.

Kumar et al. [77] buttwelded together two 5 mm thick 1/2H pure copper plates; by keeping the traverse speed constant at 50 mm/min and the rotational speeds between 600 and 800 rpm they obtained the lowest hardness values in the NZs, and at a low heat input (50 mm/min traverse speed and 400 rpm rotational speed) they obtained much higher hardness in the NZ than the base material. At the same time, Sakthivel et al. [45] made hardness measurements of pure copper butt joints with a thickness of 2 mm for which they obtained significant increases of 128–135 HV, much higher than those of the base material, i.e., 106–111 HV, by using an advance speed of 30 mm/min and a rotational speed of 1000 rpm. Kumar et al. [77] studied the influence of FSW tool-pin profile for copper, where they obtained the highest hardness value (105 HV) using a square pin and the lowest hardness value (80 HV) using a hexagonal pin, while the hardness of the base material was 110 HV, and the other tool properties and process parameters were kept the same.

Sun et al. [58] analyzed the effect of adding SiC particles with an average particle size of 5 μm in the gap between two 1/2H copper plates with a thickness of 2 mm. They obtained a uniform hardness distribution in the case of one pass of the FSW tool and a less uniform (W shape) distribution in the case of two passes of the FSW tool. In the experiment where SiC particles were not added, the joint hardness was much lower than that where the particles were added, but the mechanical strength was higher when the SiC particles were not added.

The following conclusions can be drawn from the data summarized in the above table:

- In most cases, the mechanical strength values for welded structures are comparable to or even lower than those of the base material (BM). This reduction in resistance is due to several factors, including microstructural changes that lead to material softening, the presence of microscopic defects, and the influence of processing parameters. The mechanical strength is influenced by many factors, including microstructural changes that lead to material softening, the presence of microscopic defects, and the impact of processing parameters.
- In general, the yield strength (YS) and elongation for welded structures are lower than those of the base material due to the modification of the microstructure that occurs during the FSW process.
- Hardness values are typically lower in the joint area than in the base material due to recrystallisation occurring in the joint area.
- The efficiency of FSW is typically high, with values ranging from 70% to above 100%, depending on the material and plate thickness. Welds of thin materials (less than 5 mm) tend to exhibit a higher welding efficiency, often exceeding 90%.

In the literature, it is recommended that different combinations of parameters of FSW (rotational speed and traverse speed) are adapted to the plate thickness and the quality of the copper alloy to obtain joints with mechanical properties the same as or better than the base material. Thus, for joining pure copper plates these are: Vr = 1000 rpm, Va = 160 mm/min for a 1 mm thickness using a spiral shoulder [29]; Vr = 1200 rpm, Va = 100 mm/min for a 2 mm thickness [43]; Vr = 300 rpm, Va = 250 mm/min for a 3 mm thickness [77]; Vr = 500 rpm, Va = 20 mm/min for a 4 mm thickness [59]; Vr = 400 rpm, Va = 50 mm/min for a 5 mm thickness [31]; Vr = 635 rpm, Va = 19 mm/min for a 6 mm thickness [73]. For copper alloyed plates it is recommended to use: Vr = 1000 rpm, Va = 200 mm/min for a 2 mm thickness [43]; Vr = 1600 rpm, Va = 210 mm/min for a 3 mm thickness [42]; Vr = 400 rpm, Va = 100 mm/min for a 5 mm thickness [38]; Vr = 600 rpm, Va = 50 mm/min for a 7 mm thickness [49]; Vr = 500 rpm, Va = 100 mm/min for a 10–11 mm thickness [76].

## 7. Conclusions, Bottlenecks, and Future Development Directions

This paper presents a detailed study of papers published to date regarding the FSW of pure copper and its alloys. Therefore, the quality of the joints obtained has been analyzed through the link between their microstructure and mechanical properties with the shape and material of the tool, the process parameters, and the temperature obtained during welding.

### 7.1. Conclusions

From the works published so far, it has been found that, in the friction stir welding (FSW) of copper and its alloys, it is still difficult to achieve defect-free joints with superior mechanical properties. Due to copper’s high thermal conductivity, it is challenging to maintain a constant optimal temperature in the joint area, which leads to variations in the microstructure and mechanical properties. The results indicate that, in the case of high traverse speeds and low rotational speeds, there is a significant variation in microhardness and the appearance of weak zones in the joint. These variations suggest that the process parameters and the tool geometry must be adjusted for each type of material or copper plate thickness.

The conclusions learned from the analyzed papers are presented below.

In the friction stir welding (FSW) process for pure copper and its alloys, tools with cylindrical pins (threaded or non-threaded) and with flat shoulders have frequently been used because they are simple, efficient, and due to their capacity to produce homogeneous and resisting joints when the process parameters are chosen accordingly.

High-speed steels (HSSs) are frequently used for the manufacturing of tools, although they have limitations when used for welding materials with high thermal conductivity, as copper accelerates tool wear due to the rapid dissipation of heat. However, due to their low cost, good machinability, and acceptable durability, high-speed steels are a common choice in FSW for copper.

Higher rotational and traverse speeds are used for welding thin plates (1–2 mm) because it is easier to achieve the optimal temperature. Therefore, for thin copper plates ranging from 1 to 2 mm in thickness, the optimal spindle speeds are between 800 and 1200 rpm and the traverse speeds are set at 100–200 mm/min to ensure effective heat generation without compromising joint integrity. The thicker plates usually need medium rotational speeds and traverse speeds to ensure complete penetration and superior joint quality.

During the welding process, the temperature must be well controlled to prevent the appearance of defects and to obtain a fine microstructure. For pure copper, the optimal welding temperature is around 540–550 °C. For copper alloys, the temperature can have higher values, but not more than 0.8 of the melting temperature of the material to prevent damage to the microstructure.

The nugget zone (NZ) usually presents a finer granulation than the heat-affected zone (HAZ) due to complete recrystallization, which results in a higher microhardness and tensile strength in the NZ than in the HAZ.

The size of the grains should be smaller for thin plates welded using FSW because the heat transfer is more efficient; for the welding of thicker plates, the grain size is larger, yet it can be controlled by adjusting the process parameters and using appropriate tools.

The mechanical properties in the nugget zone can be comparable to or smaller than those of the base material due to the microstructural transformations that appear during the welding process. In almost every study, the test samples exposed to traction break in the HAZ.

The copper alloys, such as Cu-Zn and Cu-Cr-Zr, present a higher microhardness and mechanical strength than pure copper because of the presence of the alloying elements which improve the material’s mechanical properties.

### 7.2. Bottlenecks

Achieving good mechanical properties in the nugget zone (NZ), similar to or surpassing those of the base material, remains challenging due to microstructural transformations that occur during welding. Most studies indicate that fractures commonly occur in the heat-affected zone (HAZ) during tensile tests, as this region is often weaker. Additionally, copper alloys like Cu-Zn and Cu-Cr-Zr tend to show increased microhardness and mechanical strength compared to pure copper, largely due to the reinforcing effects of alloying elements. However, it is difficult to maintain these enhanced properties uniformly across joints in different copper alloys, especially under varied welding conditions and process parameters.

### 7.3. Future Development Direction

Future research will explore more efficient methods for real-time temperature monitoring and control in the welding zone to achieve defect-free joints. Additionally, hybrid processes, such as tungsten inert gas (TIG), laser-assisted FSW, FSW with active cooling, or FSW in a controlled environment, could also be considered. These techniques may offer additional advantages, such as better temperature control, a reduction in defects, and improvement in the mechanical properties of the joints.

Future studies may consider the development of advanced numerical models to simulate the FSW process, including temperature-dependent material properties, heat transfer coefficients, and temperature-dependent friction coefficients. Numerical models can reduce experimentation periods and, based on them, the process can be optimized.

## Figures and Tables

**Table 1:** Particularities of tooling and FSW technological parameters of copper materials.

Material and Plate Thickness	Tool Geometry/Material	Process Parameters	Remarks	Ref.
Cu DHP R240: 1 mm	1. Flat shoulder Ø13 mm; 2. Concave shoulder Ø13 mm, 3. Spiral shoulder Ø13 mm; Cylindrical threaded pin Ø3 mm; Pin length 0.9 mm.	Vr: 400–1000 rpm; Va: 160–250 mm/min; Fa: 7 kN.	When using the flat-shouldered tool, defective joints resulted because little heat was generated and the ability to guide the material flow was poor. The best material flow, mechanical properties, and grain refinement of the NZ were achieved using the spiral-shoulder tool.	[29]
Pure Cu 1/2H: 2 mm	Concave shoulder Ø12 mm; Cylindrical pin Ø4 mm; Pin length 2 mm; Material WV alloy. Tilt angle 3°.	Vr: 200–1200 rpm; Va: 200–800 mm/min; Fa: 1000–1500 daN.	Applying a force less than 1200 daN resulted in lower hardnesses than the base material and tensile fractures occurred in the NZ, whereas for 1500 daN the hardness was higher and the specimens broke out of the embedment. At Va = 650 mm/min and Vr = 400–600 rpm and a force of 1500 daN, a refined microstructure without defects was obtained.	[13]
Cu 1/2H: 2 mm	Flat shoulder Ø12 mm; Cylindrical pin Ø4 mm; Pin length 2 mm; Material WC.	Vr: 500 rpm; Va: 100 mm/min; Fa: 1000 daN.	The addition of SiC microparticles between the contact surfaces of the plates resulted in a much finer grain structure and the joint hardness increased significantly, but the tensile strength was degraded.	[58]
Pure Cu: 3 mm	Flat shoulder Ø12 mm; Cylindrical threaded pin Ø3 mm; Pin length 1.75 mm; Material H13 tool steel.	Vr: 463–1136 rpm; Va: 16–184 mm/min; Fa: 1.66–3.34 kN.	The experimentally validated fuzzy model predicted that, for Vr = 1136 rpm, Va = 46.75 mm/min, and Fa = 3.34 kN, superior mechanical properties would be obtained. Plastic deformation has the greatest influence on grain size in the NZ. Temperatures less than 0.5 of the melting temperature led to good mechanical properties and grain cooling.	[3]
Pure Cu 1/2H: 3 mm	Concave shoulder Ø12 mm; Cylindrical threaded pin Ø3 mm; Pin length 2.5 mm; Material HSS tool steel.	Vr: 300–1000 rpm; Va: 100 mm/min.	For lower Va the cracking occurs on the advancing side, and for higher Va the cracking moves to the retreating side. Properties similar to the base material were obtained using Vr = 400 rpm. HAZ is coarse-grained compared to NZ, irrespective of the rotational speed.	[50]
Pure Cu 1/2H: 3 mm	Concave shoulder Ø12 mm; Cylindrical threaded pin Ø3 mm; Pin length 2.5 mm; Material HSS tool steel.	Vr: 600 rpm; Va: 25–200 mm/min.	Most defects were identified for lower Va. Tensile strength and joint elongation increased with increasing welding speed, reaching a maximum at Va = 50 mm/min, 100 mm/min, then decreased at higher welding speeds.	[51]
Pure Cu: 3 mm	Concave shoulder Ø14 mm; Cylindrical threaded pin Ø4 mm; Pin length 2.7 mm; Tilt angle 2.5°; Additional rapid cooling with flowing water 7 l/min.	Vr: 400–800 rpm; Va: 50 mm/min.	The joint made with Vr = 400 rpm, Va = 50 mm/min, both air cooled and rapidly water cooled, had better hardness than Vr = 800 rpm. The best mechanical properties were obtained with the water-cooled seals. All of the bearings broke in the HAZ during tensile tests.	[52]
Cu-DHP: 3 mm	Flat shoulder Ø20 mm; Conical pin Ø4–3 mm; Pin length 2.8 mm.	Vr: 800–1200 rpm; Va: 90–150 mm/min.	Average temperatures in the stability zone ranged between 466 °C and 693 °C. Increasing the rotational speed of the tool resulted in a significant increase in process temperature, while the traverse speed had less influence. Excessive burrs occurred at temperatures above 650 °C.	[27]
Pure Cu C11000: 3 mm	Flat shoulder Ø16 mm; Conical pin Ø6–4 mm; Pin length 2.7 mm; Tilt angle 2.5°.	Vr: 1000 rpm; Va: 25 mm/min; Fa: 10 kN.	This study highlights the advantages of FSW over TIG for welding pure copper. FSW joint hardness is much higher than that obtained by TIG. The grain size of NZ is the finest, followed by HAZ. Mechanical properties obtained by FSW are much better than TIG.	[44]
Cu: 3 mm	Cylindrical, conical threaded, triangular, square, pentagonal, and hexagonal pins.	Vr: 700–1100 rpm; Va: 30–50 mm/min.	The square shape of the FSW tool pin resulted in joints with very good mechanical properties. With Vr = 900 rpm and Va = 40 mm/min, the best mechanical properties are achieved.	[47]
Cu: 3 mm	Flat shoulder Ø24 mm; Cylindrical pin Ø8 mm; Pin length 2.8 mm; Material H13 tool steel.	Vr: 900–1120 rpm; Va: 25–50 mm/min.	Defect-free joints were obtained under all welding conditions. The best mechanical properties and finest grain size was obtained using Vr = 900 rpm and Va = 40 mm/min.	[48]
Pure Cu C11000: 3.1 mm	Concave shoulder Ø12 mm; Conical pin Ø3 mm; Pin length 2.8 mm; Material SKH9 high-speed steel.	Vr: 800–900 rpm; Va: 30–50 mm/min.	Defect-free joints were obtained at temperatures between 460 and 530 °C. The advancing part showed higher temperatures than the re-drawing part. The mechanical properties of the TMAZ were about 60% of those of the base material. For Vr = 900 rpm and Va = 50 mm/min and for Vr = 800 rpm and Va = 30 mm/min, good quality joints resulted, with temperatures of 530 °C and 522 °C, respectively.	[1]
Pure Cu 1/2H: 4 mm	Concave shoulder Ø12 mm; Cylindrical threaded pin Ø4 mm; Pin length 1.7 mm; Material HSS tool steel.	Vr: 200–1000 rpm; Va: 2 mm/s.	In the cases where the process temperature was below 0.5 of the melting temperature of the base material (Vr = 200–400 rpm), continuous recrystallized granular structures and a material softening were obtained, and for the cases where the temperature was above 0.5, discontinuous recrystallization was accompanied by a material softening. The hardening performed at Vr < 400 rpm (<0.5 × Tm) showed significant hardening of the material due to grain refinement.	[16]
Cu: 4 mm	Flat shoulder Ø15 mm; Cylindrical threaded pin Ø6 mm; Pin length 3.7 mm; Tilt angle 2.5°; Blown argon as a shielding gas.	Vr: 600–900 rpm; Va: 40–80 mm/min.	The HAZ was the least resistant. The NZ exhibited a fine grain size due to recrystallization, while the HAZ had a large grain size due to annealing. The corrosion resistance was improved in the NZ. The highest hardness resulted in the NZ for Vr = 600 rpm, Va = 40 mm/min, 2 passes.	[21]
Pure Cu: 4 mm	Flat shoulder Ø12 mm; Cylindrical threaded pin Ø5 mm; Pin length 3.4 mm; Material high carbon steel.	Vr: 600 rpm; Va: 45 mm/min.	Defect-free joints with 4 distinct zones were obtained, with fine grains in the NZ and coarse grains in the HAZ. Improved fatigue properties and cyclic hardening and softening behaviors were observed as a function of the strain amplitude, with cracking in the HAZ.	[28]
Pure Cu 1/2H: 5 mm	Flat shoulder Ø20 mm; Cylindrical threaded pin Ø6 mm; Pin length 4.7 mm; Tilt angle: 2.5°.	Vr: 400–800 rpm; Va: 50–200 mm/min.	The finest grain was formed in the NZ, and the HAZ had larger holes than the base material. At 400 rpm and 50 mm/min, the hardness in the NZ was significantly higher than that of the base material. The break on impact was in the HAZ for all joints.	[15]
Pure Cu: 5 mm	Spiral shoulder Ø20 mm; Cylindrical threaded pin Ø6 mm; Pin length 4.7 mm.	Vr: 400–800 rpm; Va: 50 mm/min.	The grains were smaller and the mechanical properties increased in the NZ for lower rotational speeds (400 rpm). Defect-free bearings were obtained at all rotational speeds investigated.	[31]
Pure Cu: 5 mm	Flat shoulder Ø20 mm; 1. Cylindrical threaded pin Ø6 mm; 2. Square pin 6 × 6 mm; Pin length 4.7 mm.	Vr: 600 rpm; Va: 75 mm/min.	The square-pinned tool results in finer recrystallized grains, higher temperatures, and superior mechanical properties. Using a square pine profile can significantly improve the mechanical properties of the joint.	[32]
Pure Cu: 5 mm	Flat shoulder Ø20 mm; Square pin 4.3 × 4.3 mm; Pin length 4.7 mm; Material H13 tool steel; Tilt angle: 2.5°.	Vr: 600–1200 rpm; Va: 25–100 mm/min.	High rotational speeds and a low traverse speed resulted in higher temperatures and lower cooling rates, leading to higher heat input. In the NZ the grain size was smaller and larger in the HAZ compared to the base material. The TMAZ showed an equidistant recrystallized grain structure. The NZ’s ductility showed higher values than the base material for Vr = 600 rpm and Va = 75 mm/min. Defect-free joints were obtained at high temperatures (100 mm/min, 1200 rpm) and low temperatures (25–75 mm/min, 600 rpm).	[34]
Cu: 5 mm	Flat shoulder Ø20 mm; Cylindrical threaded pin Ø8 mm; Pin length 4.7 mm.	Vr: 500–710 rpm; Va: 56–112 mm/min.	Hardness decreases with increasing temperature. Optimum welding conditions were obtained for Vr = 500 and Va = 56–112 mm/min. The HAZ showed the lowest values of mechanical properties. Charpy tests showed that the impact strength increased in all cases.	[66]
Cu: 5 mm	Flat shoulder Ø25 mm; Cylindrical pin Ø5.5 mm; Pin length 4.8 mm.	Vr: 1000 rpm; Va: 80 mm/min; Fa: 25 kN.	FSW was accompanied by an additional TIG heat source, which introduced higher process temperatures than conventional FSW. And this showed that the integration of a heat source brings significant benefits in terms of weld quality, tool wear, and process efficiency. To avoid defects, the maximum temperature should be between 70 and 80% of the melting temperature of the base material.	[57]
Cu-ETP R220: 5 mm	Spiral shoulder Ø23 mm; Conical pin Ø8–5.4 mm; Pin length 4.5 mm; Plunging speed 2.5 mm/min; Dwelling time of 5 s.	Vr: 580 rpm; Va: 40–80 mm/min.	The mechanical properties of the joints were significantly lower than those of the base material. The elastic behavior decreased by 60% and the maximum stress by 15% of those of the base material due to excessive heating (800 °C). Joints welded at 60 mm/min exhibited the best mechanical properties, including hardness, tensile strength, and fatigue strength.	[55]
Pure Cu: 6 mm	Flat shoulder Ø15 mm; Conical threaded pin Ø6–4 mm; Pin length 5.6 mm; Material H13 tool steel.	Vr: 635 rpm; Va: 8–19 mm/min.	The joint hardnesses were higher than those of the base material. For Va = 19 mm/min, joints with mechanical properties superior to the base material were obtained.	[73]

**Table 2:** Particularities of tooling and FSW technological parameters of copper alloy materials.

Material and Plate Thickness	Tool Geometry/Material	Process Parameters	Remarks	Ref.
Pure Cu–Cu-Zn30%: 2 mm	Flat shoulder Ø12 mm; Cylindrical pin Ø3 mm; Pin length 1.75 mm; Material H13 tool steel.	Vr: 400 rpm; Va: 100 mm/min.	The NZ has a microstructure with small holes. The simultaneous presence of heat and plastic deformation resulted in joints with improved mechanical properties.	[30]
Pure Cu–Cu-Zn30%: 2 mm	Flat shoulder Ø12 mm; Cylindrical pin Ø3 mm; Pin length 1.75 mm; Material H13 tool steel.	Vr: 563–1236 rpm; Va: 33–117 mm/min; Fa: 1.16–2.8 kN.	Maximum hardness can be obtained using Vr = 700 rpm, Va = 100 mm/min, and Fa = 1.5 kN. The parameter with the greatest effect on hardness was the rotational speed, followed by axial force and traverse speed.	[35]
Cu-Cr-Zr: 2 mm	Flat shoulder Ø18 mm; Cylindrical pin Ø6 mm; Pin length 1.8 mm; Material H13 tool steel; Laser Shock Peening.	Vr: 400–800 rpm; Va: 50–150 mm/min; Energy: 0.8 J; Frequency: 10 Hz.	The NZ shows higher mechanical properties and elongations longer than the base material, and for the HAZ they are similar to those of the base material. Optimum parameters: Vr = 600 rpm, Va = 100 mm/min. LSP significantly improved the mechanical and microstructural properties of the base material and CuCrZr FSW welds.	[36]
Cu-Zn: 2 mm	Flat shoulder Ø12 mm; Cylindrical threaded pin Ø3 mm; Pin length 1.75 mm; Material H13 tool steel.	Vr: 463–1136 rpm; Va: 16–184 mm/min; Fa: 1.66–3.34 kN.	Rotational speed had the greatest effect on hardness, followed by axial force and traverse speed. Increasing Vr and Fa or decreasing Va decreased the hardness of the joints due to increased grain size. Maximum hardness was obtained for Vr = 629,2 rpm, Va = 107.2 mm/min and Fa = 2.3 kN.	[39]
Pure Cu, Cu-Zn30%, Cu-Zn37%: 2 mm	Flat shoulder Ø12 mm; Cylindrical pin Ø3 mm; Pin length 1.7 mm; Material H13 tool steel.	Vr: 450–900 rpm; Va: 100–300 mm/min.	Higher Zn content led grains to shrink and hardness increased. Higher Vr and lower Va caused an increase in grain size and a decrease in hardness. The developed mathematic models were adequate and accurately predicted grain size and joint hardness. For Vr = 450 rpm and Va = 300 mm/min joints, the best mechanical properties were obtained.	[53]
Cu-Zn40%: 2 mm	Flat shoulder Ø12 mm; Cylindrical pin Ø4 mm; Pin length 2 mm; Tilt angle: 2.5°.	Vr: 250–1500 rpm; Va: 500–2000 mm/min.	Joints made with higher Vr showed extremely fine grains in the NZ and TMAZ. Hardnesses ranging from 1.4 to 1.7 of the base material were obtained in the NZ, and for all joints this zone also exhibited the highest stresses. Defect-free joints were obtained in a relatively wide range of welding conditions: Vr = 1000–1500 rpm and Va = 500–2000 mm/min.	[41,72]
Pure Cu, Cu-Zn30%: 2 mm	Flat shoulder Ø10 mm; Cylindrical threaded pin Ø3 mm; Pin length 1.3 mm; Material H13 tool steel; Conducted under cold flowing water.	Vr: 1200 rpm; Va: 100–200 mm/min.	Very good strengths and ductility were obtained in the NZs of both materials. The microstructure exhibited ultrafine grains and low dislocation densities. The bond zone showed improved tensile strength and hardness compared to the base materials.	[43]
Cu-Cr-Zr: 2.5 mm	1. Concave shoulder Ø12 mm; Material TiC based cermet; 2. Flat shoulder Ø12 mm; Conical threaded pin Ø5 mm; Pin length 2.3 mm; Material W-Re (Wolfram-Rhenium).	Vr: 400–600 rpm; Vr: 600 rpm for W-Re Va: 50 mm/min.	NZ grains became smaller with decreasing Vr. Strength close to that of the base material was identified using Vr = 400 rpm. Welding with the W-Re tool at Vr = 600 rpm led to the formation of defects in the joint, with a negative impact on the mechanical properties and electrical conductivity.	[67]
Cu-Zn30%: 3 mm	Flat shoulder Ø13 mm; Cylindrical threaded pin M5 × 1.5 mm; Pin length 2.9 mm; Material X32CrMoV12-28.	Vr: 2050 rpm; Va: 20–140 mm/min.	Surface defects and tunnel-type defects were encountered. Properties close to those of the base material were obtained by using Va = 112 mm/min.	[6]
Cu-Cr-Zr: 3 mm	Flat shoulder Ø20 mm; Conical pin Ø5–3 mm; Pin length 2.7 mm.	Vr: 800–1200 rpm; Va: 40–100 mm/min.	The degree of recrystallization and grain size growth in the NZ had a direct correlation with Vr and an inverse correlation with Va. The tensile strength was about 60% of that of the base material under aging, but higher under annealed conditions. The joints performed at rotation speeds between 800 and 1200 rpm and traverse speeds between 40 and 100 mm/min were defect-free.	[8]
Cu-Zn30%: 3 mm	Concave shoulder Ø12 mm; Cylindrical threaded pin Ø4 mm; Pin length 1.7 mm.	Vr: 200–600 rpm; Va: 2 mm/s.	The experiments achieved temperatures between 0.46 and 0.8 of the melting temperature of the base material. In all cases, the grain sizes were smaller in the NZ, which was also the hardest zone. Optimum Vr: 300–600 rpm.	[40]
Cu-Zn10%, Cu-Zn30%: 3 mm	Conical pin; Material X32CrMo3 3.	Vr: 1600 rpm; Va: 160–260 mm/min.	In the case of higher Zn content, porosities were formed in the NZ for all Va values. The highest strength and ductility were obtained for Va = 210 mm/min.	[42]
Cu-Cr-Zr-Ti: 3.8 mm	Flat shoulder Ø15 mm; Conical threaded pin Ø8–6 mm; Pin length 3 mm; Material Inconel 718.	Vr: 1000 rpm; Va: 30 mm/min; Fa: 10 kN.	The TMAZ showed recrystallized and partially recrystallized fine grains. The flow resistance of the joint was 131% of that of the base material.	[65]
Cu (0.7 Ni-0.3 Cr-0.12 Fe-0.04 Ti): 4 mm	Concave shoulder Ø17.8 mm; Conical pin Ø6.5–5.5; Pin length 3.5 mm; The shoulder material T6 grade of tungsten high-speed steel; The pin material C-2 grade of sintered carbide.	Vr: 710 rpm; Va: 40 mm/min.	Voids and cracks were observed on the feed side, caused by low heat input and insufficient material deposition behind the tool. Precise control of the penetration depth can prevent defects and improve the quality of the weld, ensuring uniform material distribution and the formation of a defect-free weld layer.	[56]
Cu-Zn37%: 4 mm	Flat shoulder Ø20 mm; Cylindrical threaded pin M6 × 1 mm; Pin length 3.8 mm; Material X210Cr12.	Vr: 750–1000 rpm; Va: 20–60 mm/min.	NZ and TMAZ grain sizes decreased with increasing Va. For Vr = 100 rpm and Va = 20 mm/min, tunnel-type defects appeared. The fatigue life was higher by using Vr = 750 rpm and Va = 20 mm/min. In all cases the fatigue failure was located in the retraction side.	[64]
Cu (0.7 Ni-0.3 Cr-0.12 Fe-0.04 Ti): 4 mm	Concave shoulder Ø17.8 mm; Conical pin Ø6.5–5.5; Pin length 3.5 mm; The shoulder material T6 grade of tungsten high-speed steel; The pin material C-2 grade of sintered carbide.	Vr: 500–710 rpm; Va: 20–63 mm/min.	At Vr = 710 rpm and Va = 40 mm/min a good tensile strength was obtained, i.e., a good quality joint without major defects. Onion ring structures could be observed. By increasing the traverse speed or reducing the rotational speed, the mechanical properties were increased and the slip decreased.	[59]
Cu-Zn 1/2H: 5 mm	Flat shoulder Ø18 mm; Cylindrical threaded pin Ø6 mm; Pin length 4.7 mm; Tilt angle: 2.5°.	Vr: 400–1000 rpm; Va: 100 mm/min.	Grain recrystallization was incomplete in the NZ. The maximum stresses of the joints were very close to those of the base material, and breakage occurred in the HAZ. The best results were obtained for Vr = 600 rpm.	[38]
Cu-Zn 1/2H: 5 mm	Flat shoulder Ø18 mm; Cylindrical threaded pin Ø6 mm; Pin length 4.7 mm; Tilt angle 2.5°.	Vr: 600 rpm; Va: 100 mm/min.	The upper surface and the leading part of the NZ showed fully recrystallized fine grains, while partial dynamic recrystallization was observed in the rest of the bead. The joint zone exhibited increased hardness due to grain refinement and joint cracking occurred in the HAZ.	[54]
Cu-Cr-Zr: 7 mm	Concave shoulder Ø20 mm; Conical threaded pin Ø8 mm; Pin length 6.8 mm; Material Ni-base; 1. Solid solution treatment 980 °C for 1.5, followed by rapid water cooling; 2. Peak-aged, 480 °C, 2 h.	Vr: 300–600 rpm; Va: 50–100 mm/min.	The strength was lower than that of the base material for the aged joint and higher for the water-cooled joint. For Vr = 600 rpm and Va = 50 mm/min (welding temperature 481 °C) the best mechanical strength and electrical conductivity resulted due to efficient temperature control and the use of water cooling.	[49]

**Table 3:** Temperature during the welding process.

Material and Plate Thickness	Tool Geometry	Process Parameters	Measuring Procedure	Measuring Zone	Value	Ref.
Cu-DHP: 3 mm	Flat shoulder; Conical pin.	Vr: 800 rpm; Va: 150 mm/min.	Infrared thermographic camera	Constant distance from the welding zone, continuously monitoring the temperature near the tool.	500 °C	[63]
Cu-Cr-Zr: 3 mm	Flat shoulder; Conical pin.	Vr: 800 rpm; Va: 40 mm/min.	Thermocouples	At 2 mm from the shoulder.	≈750 °C	[8]
Cu-Zn30%: 3 mm	Concave shoulder; Cylindrical conical pin;	Vr: 600 rpm; Va: 2 mm/s.	Thermocouples	Integrated directly into the workpiece and positioned so as to be destroyed by the passage of the tool.	≈700 °C	[40]
Pure Cu C11000: 3.1 mm	Flat shoulder; Conical pin.	Vr: 800; 900 rpm; Va: 30; 50 mm/min.	Thermocouples	At 6 mm from the joint line, on both the forward (AS) and rear (RS).	460–530 °C	[1]
Pure Cu 1/2H: 4 mm	Concave shoulder; Cylindrical conical pin;	Vr: 500 rpm; Va: 2 mm/s.	Thermocouples	Integrated directly into the workpiece and positioned so as to be destroyed by the passage of the tool.	≈550 °C (0.5 × Tm)	[16]
Pure Cu: 4 mm	Concave shoulder; Conical pin.	Vr: 710 rpm; Va: 40 mm/min.	Thermocouples, FEM analysis	At 6 mm from the line of trim and at a depth of 2 mm from the upper surface, on (AS).	≈500 °C	[74]
Pure Cu: 5 mm	Flat shoulder; 1. Cylindrical conical pin; 2. Square pin.	Vr: 600 rpm; Va: 75 mm/min.	Thermocouples	Close to the finishing line at several points.	1. ≈ 380 °C 2. ≈ 450 °C	[32]
Pure Cu: 5 mm	Flat shoulder; Square pin.	Vr: 1200 rpm; Va: 100 mm/min.	Thermocouples	Close to the joint line, 120 mm from the end of the plate.	492 °C	[34]
Cu-ETP R220: 5 mm	Spiral shoulder; Conical pin.	Vr: 580 rpm; Va: 60 mm/min.	Thermal imaging camera	On the surface of the plate near the shoulder.	≈600 °C	[55]
Cu-Cr-Zr: 7 mm	Concave shoulder; Conical threaded pin.	Vr: 600 rpm; Va: 50 mm/min.	K-type thermocouples	At the edge of the NZ.	481 °C	[49]

**Table 4:** Macroscopic aspect of the joint at different temperatures [63].

Rotation Rate [rpm]	Traverse Speed [mm/min]	Temperature [°C]	Morphological Surface Joints
1200	90	693	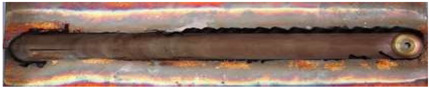
1000	90	466	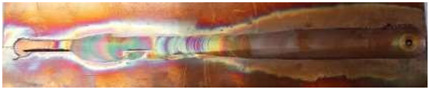
1200	150	580	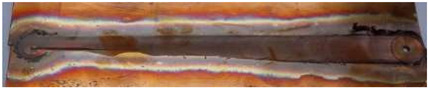
1000	150	550	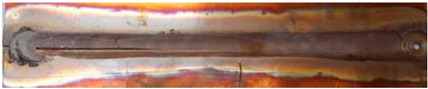
800	90	476	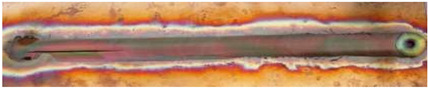
800	150	500	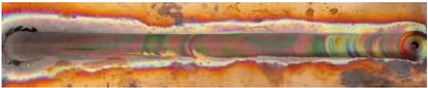
1000	120	596	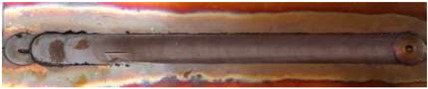

**Table 5:** Grain size of Cu and Cu alloys in the nugget zone (NZ).

Material and Plate Thickness [mm]	Tool Geometry	Rotation Rate [rpm]	Traverse Speed [mm/min]	Grain Size [μm]	Ref.
Cu-DHP R240: 1	Cylindrical threaded pin	400–1000	160–250	1–8	[29]
Pure Cu–Brass: 2	Cylindrical pin	400	100	2.7–3.6	[30]
Cu-Zn: 2	Cylindrical pin	563–1236	33–117	6.6–19.8	[35]
Cu-Cr-Zr: 2	Cylindrical pin	400–800	50–150	1.45	[36]
Pure Cu 1/2H: 2	Cylindrical pin	200–1200	200–800	3.77–24.1	[13]
Cu-Zn: 2	Cylindrical threaded pin	463–1136	16–184	4.2–15.6	[39]
Pure Cu, Cu-Zn30%, Cu-Zn37%: 2	Cylindrical pin	450–900	100–300	1.6–17.1	[53]
Cu-Zn40%: 2	Cylindrical pin	250–1500	500–2000	0.8–22.5	[41,72]
Pure Cu, Cu-Zn30%: 2	Cylindrical threaded pin	1200	100–200	0.98; 0.54	[43]
Cu: 2	–	1000	30	11	[45]
Cu 1/2H: 2	Cylindrical pin	500	100	2–8	[58]
Cu-Cr-Zr: 2.5	Conical threaded pin	400–600	50	4.5–8.2	[67]
Pure Cu: 3	Cylindrical threaded pin	463–1136	16–184	46	[3]
Pure Cu 1/2H: 3	Cylindrical threaded pin	300–1000	100	4	[50]
Pure Cu 1/2H: 3	Cylindrical threaded pin	600	25–200	4.27–5.26	[51]
Cu-Cr-Zr: 3	Conical pin	800–1200	40–100	3–20	[8]
Cu-Zn30%: 3	Cylindrical threaded pin	200–600	120	0.4–3	[40]
Cu-Zn10%, Cu-Zn30%: 3	Conical pin	1600	160–260	0.3	[42]
Pure Cu C11000: 3	Conical pin	1000	25	110.5–150	[44]
Cu: 3	Cylindrical pin	300	50–250	3–9.3	[75]
Cu-Cr-Zr-Ti: 3.8	Conical threaded pin	1000	30	65 ± 9	[65]
Cu: 4	Cylindrical threaded pin	600–900	40–80	2.3–16.1	[21]
Cu T2: 4	Cylindrical pin	600–1200	50	22.51–57.61	[7]
Pure Cu 1/2H: 4	Cylindrical threaded pin	200–1000	2	1–30	[16]
Pure Cu: 5	Cylindrical threaded pin/square pin	600	75	10–15	[32]
Cu: 5	Cylindrical threaded pin	500–710	56–112	2.4–17.3	[66]
Cu-ETP R220: 5	Conical pin	580	40–80	10	[55]
Pure Cu: 5	–	600–900	25–75	9–14	[46]
Pure Cu 1/2H: 5	Cylindrical threaded pin	400–800	50–200	3–16	[15]
Pure Cu: 5	Cylindrical threaded pin	400–800	50	3.5–12	[31]
Pure Cu: 6	Conical threaded pin	635	8–19	12.59–15.62	[73]
Cu-Cr-Zr: 7	Conical threaded pin	300–600	50–100	20	[49]
Cu-Cr-Zr: 12	–	–	–	10–15	[76]

**Table 6:** Mechanical properties of Cu and Cu alloy FSW joints.

Material and Plate Thickness [mm]	Tool Geometry	Process Parameters	UTS [MPa]/YS [MPa]	Microhardness [HV]	Elongation [%]	Joint Efficiency [%]	Ref.
Cu DHP R240: 1	Cylindrical threaded pin	Vr: 400–1000 rpm; Va: 160–250 mm/min.	BM: 320 FSW: 287–325	BM: 92 FSW: 90–130 *	–	90–102	[29]
Cu T2: 2	Cylindrical pin	Vr: 600–1200 rpm; Va: 50 mm/min.	FSW: 190–233.25/65.04–137.45	FSW: 75–85 *	FSW: 8.84–47.64	–	[7]
Pure Cu 1/2H: 2	Cylindrical pin	Vr: 200–1200 rpm; Va: 200–800 mm/min.	BM: 266/194 FSW: 225–266/101–255	BM: 100 * FSW: 65–103 *	BM: 47 FSW: 20–47	85–100	[13]
Pure Cu: 2	Cylindrical threaded pin	Vr: 1200 rpm; Va: 100–200 mm/min.	BM: 210 */70 * FSW: 310 */275	BM: 71.3 FSW: 108.4	–	68	[43]
Cu: 2	-	Vr: 1000 rpm; Va: 30 mm/min.	BM: 273 FSW: 231	BM: 101–111 FSW: 128–136	BM: 3.1 FSW: 1.2	85	[45]
Cu 1/2H: 2	Cylindrical pin	Vr: 500 rpm; Va: 100 mm/min.	FSW: 125–228	FSW: 70–110	–	–	[58]
Pure Cu C11000: 3	Conical pin	Vr: 1000 rpm; Va: 25 mm/min.	BM: 212/68 FSW: 194/70	BM: 95 * FSW: 80 *	BM: 28.1 FSW: 22.8	92	[44]
Cu: 3	Cylindrical, conical threaded, triangular, square, pentagonal, and hexagonal pins	Vr: 700–1100 rpm; Va: 30–50 mm/min.	FSW: 158.29–212.07/90.88–167.06	FSW: 77.89–98.94	FSW: 3.01–15.09	–	[47]
Cu: 3	Cylindrical pin	Vr: 900–1120 rpm; Va: 25–50 mm/min.	BM: 260/231 FSW: 121–168/95–112	BM: 110 FSW: 62–85	BM: 31 FSW: 8.8–13.5	47–65	[48]
Pure Cu 1/2H: 3	Cylindrical threaded pin	Vr: 600 rpm; Va: 25–200 mm/min.	FSW: 258–272 *	FSW: 88–108 *	FSW: 1.5–11.5 *	–	[51]
Pure Cu: 3	Cylindrical threaded pin	Vr: 400–800 rpm; Va: 50 mm/min.	BM: 350/330 FSW: 220–340/100–315	BM: 115 FSW: 70–105 *	BM: 9 FSW: 8–21	63–97%	[52]
Cu: 3	Cylindrical pin	Vr: 300 rpm; Va: 50–250 mm/min.	BM: 270/209 FSW: 319–328/236–261	BM: 84.6 FSW: 101.9–113.6	BM: 22 FSW: 23–24	118–121	[75]
Cu: 3	Cylindrical, conical threaded, triangular, square, pentagonal, and hexagonal pins	Vr: 900 rpm; Va: 40 mm/min.	BM: 260/231 FSW: 168–218/109–182	BM: 110 FSW: 85–105	BM: 31 FSW: 3–16	65–85	[77]
Pure Cu C11000: 3.1	Conical pin	Vr: 800–900 rpm; Va: 30–50 mm/min.	BM: 282 * FSW: 192–219 *	BM: 104 * FSW: 75–85*	–	68–78	[1]
Cu: 4	Cylindrical threaded pin	Vr: 600–900 rpm; Va: 40–80 mm/min.	BM: 352/151 FSW: 249–346/117–146	BM: 91 FSW: 107–130 *	–	71–98	[21]
Cu: 4	Conical pin	Vr: 500–710 rpm; Va: 20–63 mm/min.	BM: 301.6/232 FSW: 290–368/144–331	BM: 120–130 FSW: 105–155	BM: 18.9 FSW: 4–18	96–122	[59]
Pure Cu 1/2H: 5	Cylindrical threaded pin	Vr: 400–800 rpm; Va: 50–200 mm/min.	BM: 236.7/222.9 FSW: 228.7–236.3/122.1–207.7	BM: 81 * FSW: 62–99 *	BM: 27.7 FSW: 15.1–24.7	97–100	[15]
Pure Cu: 5	Cylindrical threaded pin	Vr: 400–800 rpm; Va: 50 mm/min.	BM: 236 */224 * FSW: 239–240 */142–174 *	BM: 82.2 FSW: 63.1–99.6	BM: 28 * FSW: 39–45 *	101	[31]
Pure Cu: 5	Cylindrical threaded pin/square pin	Vr: 600 rpm; Va: 75 mm/min.	BM: 234/178 FSW: 202–221/115–134	BM: 107 FSW: 87–99	–	86–94	[32]
Pure Cu: 5	Square pin	Vr: 600–1200 rpm; Va: 25–100 mm/min.	FSW: 222–267 */114–127 *	BM: 74 * FSW: 63–87 *	FSW: 35–44 *	–	[34]
Pure Cu: 5	Conical threaded pin	Vr: 300–600 rpm; Va: 50–100 mm/min.	BM: 234/178 FSW: 216–221/118–127	BM: 107 FSW: 81–88	BM: 47 FSW: 36–43	92–94	[49]
Cu-ETP R220: 5	Conical pin	Vr: 580 rpm; Va: 40–80 mm/min.	BM: 252.7–262.2/232–242 FSW: 209–220.7/82–101.3	BM: 94 * FSW: 77–82 *	BM: 67.3–69.2 FSW: 40.3–70.5	83–85	[55]
Cu: 5	Cylindrical threaded pin	Vr: 700–710 rpm; Va: 56–112 mm/min.	BM: 331/137 FSW: 235–326/106–134	BM: 77 * FSW: 90–110 *	BM: 29 FSW: 31–38	71–98	[66]
Pure Cu: 6	Conical threaded pin	Vr: 635 rpm; Va: 8–19 mm/min.	BM: 273.2/240.7 FSW: 138.8–256.9/93.8–165.4	BM: 97	BM: 33.54 FSW: 5.51–24.44	58–107	[73]
CuCrZr: 2	Cylindrical pin	Vr: 400–800 rpm; Va: 50–150 mm/min.	BM: 244–250 */174–230 * FSW: 230–240 */168–225 *	BM: 95–114 * FSW: 121–135	BM: 34–39 * FSW: 25–28 *	94–96	[36]
Cu70%-Zn30%: 2	Cylindrical pin	Vr: 450 rpm; Va: 100 mm/min.	BM: 230 * FSW: 300 *	BM: 90 * FSW: 125 *	–	130	[37]
Cu60%-Zn40%: 2	Cylindrical pin	Vr: 250–1500 rpm; Va: 500–2000 mm/min.	BM: 380 */195 * FSW: 370–385 */190–210 *	BM: 100 * FSW: 130–160 *	BM: 60 * FSW: 21–51 *	97–101	[41]
Cu-Zn30: 2	Cylindrical threaded pin	Vr: 1200 rpm; Va: 100–200 mm/min.	BM: 360 */110 * FSW: 490 */408	BM: 96.7 FSW: 155.2	–	136	[43]
Cu-Cr-Zr: 2	Conical threaded pin	Vr: 400–600 rpm; Va: 50 mm/min.	BM: 488/437 FSW: 382–488/320–439	BM: 151 FSW: 107–173	BM: 17.6 FSW: 8.9–11.8	78–100	[67]
CuZn30%: 3	Cylindrical threaded pin	Vr: 2050 rpm; Va: 20–140 mm/min.	BM: 360/215 FSW: 175–340/130–210	BM: 95 * FSW: 155 *	BM: 67	49–94	[6]
Cu-Zn: 3	Cylindrical threaded pin	Vr: 463–1136 rpm; Va: 16–184 mm/min.	FSW: 272–319	FSW: 90–135	FSW: 27–56	–	[39]
Cu90%-Zn10%: 3	Conical pin	Vr: 1600 rpm; Va: 160–260 mm/min.	BM: 249/139 FSW: 233–252/137–143	BM: 75 FSW: 110 *	BM: 19 FSW: 11–16	94–101	[42]
Cu70%-Zn30%: 3	Conical pin	Vr: 1600 rpm; Va: 160–260 mm/min.	BM: 341/217 FSW: 329–400/166–283	BM: 82 FSW: 85 *	BM: 17 FSW: 3–14	96–117	[42]
Cu-Cr-Zr-Ti: 3.8	Conical threaded pin	Vr: 1000 rpm; Va: 30 mm/min.	BM: 240/74 FSW: 221–272 */64–171	BM: 70 * FSW: 65–85 *	BM: 38 FSW: 13–42	92–113	[65]
Cu-Zn 1/2H: 5	Cylindrical threaded pin	Vr: 400–1000 rpm; Va: 100 mm/min.	BM: 400 */230 * FSW: 400 */180–190 *	BM: 110 * FSW: 123–136 *	BM: 50 * FSW: 39–46 *	100	[38]
Cu-Cr-Zr: 7	Conical threaded pin	Vr: 300–600 rpm; Va: 50–100 mm/min.	BM: 239–437 FSW: 240–437	BM: 80–135.8 FSW: 89–152.1	–	71–120	[49]
Cu-OF: 10–11	Pin triflate	Vr: 1400 rpm; Va: 600 mm/min.	BM: 298/292 FSW: 217/120–128	BM: 100 * FSW: 65 *	BM: 13 FSW: 38	73	[78]
Cu-DHP: 10–11	Pin triflate	Vr: 1000–1125 rpm; Va: 200–400 mm/min.	BM: 208/66 FSW: 215–230/103–115	BM: 70 * FSW: 67–73 *	BM: 56 FSW: 44–50	103–111	[78]
CuAl5Zn5Sn: 10–11	Pin triflate	Vr: 350–500 rpm; Va: 70–100 mm/min.	BM: 433/170 FSW: 439–443/175–195	BM: 90 * FSW: 105–130 *	BM: 71 FSW: 59–66	101–102	[78]
Cu-Cr-Zr: 12	-	-	BM: 449.736 FSW: 266.451	BM: 136–148 FSW: 88–99	BM: 29.733 FSW: 25.412	59	[76]
Cu-Cr-Zr: 21	Conical pin	Vr: 1500 rpm; Va: 150 mm/min.	BM: 450 * FSW: 260–280	BM: 150 * FSW: 65–80 *	BM: 12 * FSW: 16–21 *	57–62	[14]
Cu-Cr-Zr: 30	Conical threaded pin	Vr: 1500 rpm; Va: 30 mm/min.	BM: 450 */400 * FSW: 260–280 */205–230 *	BM: 150 * FSW: 65–82 *	BM: 13 * FSW: 16–21 *	58–62	[33]

* Values recovered from diagrams or graphs.

## Data Availability

The corresponding author will provide the data used in this work upon reasonable request.
